# Success of Anterior Ethmoidal Artery Flaps for Nasal Septal Perforation Repair: A Systematic Review

**DOI:** 10.1002/lary.70719

**Published:** 2026-07-01

**Authors:** Hemali P. Shah, Ethan Tsao, Alhan Sayyed, C. Scott Dorris, John R. Craig, Daniel B. Spielman

**Affiliations:** ^1^ Department of Otolaryngology—Head & Neck Surgery MedStar Georgetown University Hospital Washington District of Columbia USA; ^2^ Georgetown University School of Medicine Washington District of Columbia USA; ^3^ Dahlgren Memorial Library Georgetown University School of Medicine Washington District of Columbia USA; ^4^ Department of Otolaryngology‐Head and Neck Surgery Henry Ford Health Detroit Michigan USA

**Keywords:** nasal septal perforation, reconstruction, septal perforationanterior ethmoid artery flap, transnasal endoscopic surgery

## Abstract

**Objective:**

Nasal septal perforation (NSP) repair using the anterior ethmoidal artery (AEA) flap is an emerging endoscopic technique. This systematic review aimed to provide a comprehensive overview of the AEA flap technique, its variations, and its efficacy in repairing NSPs.

**Data Sources:**

MEDLINE, Embase, CENTRAL, and Web of Science were searched. This study was reported per the Preferred Reporting Items for Systematic Reviews and Meta‐Analyses guidelines from 1946 to 2026.

**Review Methods:**

The aforementioned search yielded 2838 results. Animal and cadaver studies, editorials, abstracts, and studies that did not address NSP repair with surgical flaps were excluded. After full‐text review of 164 studies, 16 were included.

**Results:**

A total of 249 patients were captured (223 adults, 26 pediatric). For adults, 175 patients underwent the original AEA flap technique with (*N* = 74) or without (*N* = 101) an interposition graft, such as tragal cartilage, acellular dermal, or porcine collagen grafts. All pediatric and 48 adult patients underwent various modified AEA flap techniques. For the original AEA flap, mean reported NSP size ranged from 12.8 to 22 mm and postoperative follow‐up time ranged from 3 to 51 months. Overall, NSP closure success rate was 84.6% (*N* = 148/175) for the AEA flap. Closure success rate was 83.8% (*N* = 62/74) for those with an interposition graft and 85.1% (*N* = 86/101) for patients without a graft (*p* = 0.81, Cramer's *V* = 0.02).

**Conclusions:**

With an overall closure success rate of 84.6%, the AEA flap is a reliable endoscopic technique for repairing NSPs < 2.5 cm. Emerging AEA flap modifications may further improve outcomes, while enabling repair of larger perforations.

**Level of Evidence:**

N/A.

## Graphical abstract

Nasal septal perforation (NSP) repair using the anterior ethmoidal artery (AEA) flap is an emerging endoscopic technique with an overall closure success rate of 84.6%. The AEA flap is a reliable endoscopic technique for repairing NSPs < 2.5 cm. Emerging AEA flap modifications may further improve outcomes, while enabling repair of larger perforations.

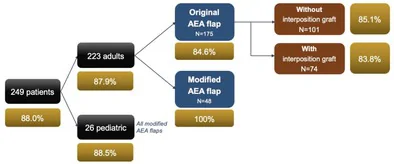

## 1 Introduction

Nasal septal perforations (NSPs) are relatively common with a reported prevalence of 1%–2% in the general population [1, 2]. While NSPs are most commonly caused by iatrogenic and physical trauma, numerous other causes include intranasal substance use, inhalants, neoplasms, infections, and autoimmune diseases [3]. NSPs can be characterized by their size, and anterior or posterior location. Anterior NSPs are more likely to be symptomatic compared to posterior NSPs due to greater disruption of nasal laminar airflow [4]. Patients with symptomatic NSPs present with crusting, epistaxis, whistling, and nasal obstruction. While the majority of patients with NSPs can be managed adequately with various topical moisturizing therapies, patients with persistent symptoms can benefit from surgical NSP repair.

Surgical repair of symptomatic NSPs aims to close the defect with minimal tension and restore nasal function. Repair can be challenging depending on the NSP size, location, and etiology. Numerous surgical options for NSP repair have been reported [5]. Open approaches may involve rhinoplasty and the use of local mucosal flaps, regional pedicled flaps, free fascial grafts, autologous cartilage grafts, and microvascular free tissue transfers [3, 6]. Endoscopic NSP repair has become increasingly popular in recent years because it yields similar high success rates to open approaches, but with potentially lower morbidity [3, 5, 7]. Numerous endoscopic NSP repair options have been described, varying by tissue type used for closure (unilateral versus bilateral and pedicled versus random mucosal flaps, free mucosal grafts, synthetic grafts), number of layers of repair (1–3 layers), and interposition grafting source (none, autologous, synthetic) [5]. Success rates of endoscopic NSP repair range from 70% to 90, and surgical efficacy may depend on NSP characteristics, patient factors, and surgeon experience [3, 5, 7, 8].

The anterior ethmoidal artery (AEA) flap is an endoscopic technique that was first reported by Castelnuovo et al. in 2011 [9]. The AEA flap was initially described as a unilateral, presumably axial mucosal flap based on septal branches from the AEA. The flap is robust, comprises nasal septal and floor mucosa, and is readily transposed via its superiorly based pedicle or base [3, 9]. Since the AEA flap's inception, several authors have published success rates of the AEA flap as well as technique for NSP repair. However, most reports are limited to case series or small retrospective cohort studies. This systematic review aimed to synthesize and analyze all available data on NSP repair success for both the AEA flap and certain AEA flap technique modifications. The primary outcome measure was successful NSP closure with the AEA flap.

## 2 Materials and Methods

A comprehensive literature search related to surgical repair of NSPs and anterior ethmoidal artery (AEA) flaps was performed. This study was reported as per the Preferred Reporting Items for Systematic Reviews and Meta‐Analyses (PRISMA) guidelines. To capture all pertinent studies, key terms for NSPs and surgical flaps for repair were implemented by an academic librarian to perform the initial search string (Supporting Information); the search strategy was not peer‐reviewed. The following data sources were searched: MEDLINE, Embase, CENTRAL, and Web of Science. Although the AEA flap was initially described by Castelnuovo in 2011, the aforementioned databases were searched from database inception to the present time (1946 to March 2026) to ensure no previous allusions to the AEA flap were missed.

All identified records were imported into the Covidence systematic review software for screening, removing duplicates, and final selection by the review team. Duplicates were identified and removed (Figure 1). Title and abstract screening was performed independently by two reviewers (E.T. and A.S.) with inter‐rater reliability quantified by Cohen's kappa = 0.46. Discrepancies and disagreements between the two primary reviewers were resolved independently by a third reviewer (H.P.S.). Exclusion criteria included non‐English or animal studies, editorials, conference abstracts, and studies that did not address NSP repair with surgical flaps.

**FIGURE 1:**
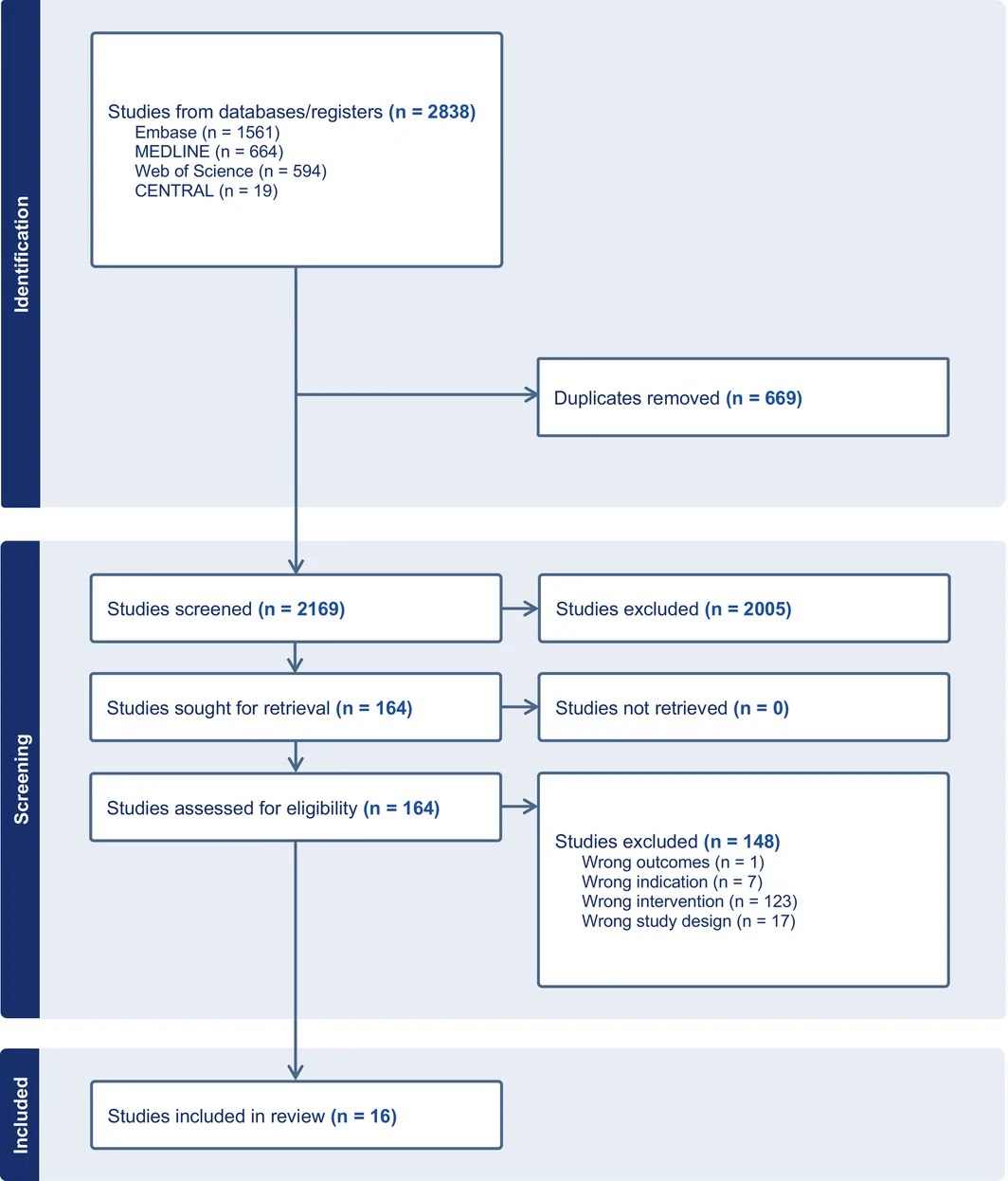
Preferred Reporting Items for Systematic Reviews and Meta‐Analyses (PRISMA) diagram outlining the search process and selection of manuscripts. [Color figure can be viewed in the online issue, which is available at www.laryngoscope.com]

Full‐text analysis was performed by three authors (E.T., A.S., and H.P.S.) to identify studies on original or modified AEA flaps for NSP repair. The original AEA flap was defined by the technique initially described by Castelnuovo et al. (Figure 2). AEA flaps that incorporated any flap modification upon the original technique were categorized as modified AEA flaps. Modified AEA flap techniques included extended AEA flaps, variations in tunneling and inset, etc. Studies were excluded if they did not report original data on the use of an AEA‐based flap for NSP repair, or if they reported the use of AEA flaps for indications other than NSP repair (e.g., skull base reconstruction, re‐mucosalization of sinus ostium). Two cadaveric studies without any patient data were also excluded. Reference lists of full‐text articles were also reviewed for additional studies, but none met inclusion criteria. Studies were categorized as retrospective cohort studies if patients were sampled based on exposures that were compared (e.g., groups with different flap techniques); studies were otherwise classified as case series with outcomes‐based sampling. Overall quality of articles was assessed using the MINORS (Methodological items for non‐randomized studies) criteria and the Oxford Centre for Evidence‐Based Medicine Level of Evidence [10, 11]. Bias was determined using the National Institute of Health's study quality assessment tool [12].

**FIGURE 2:**
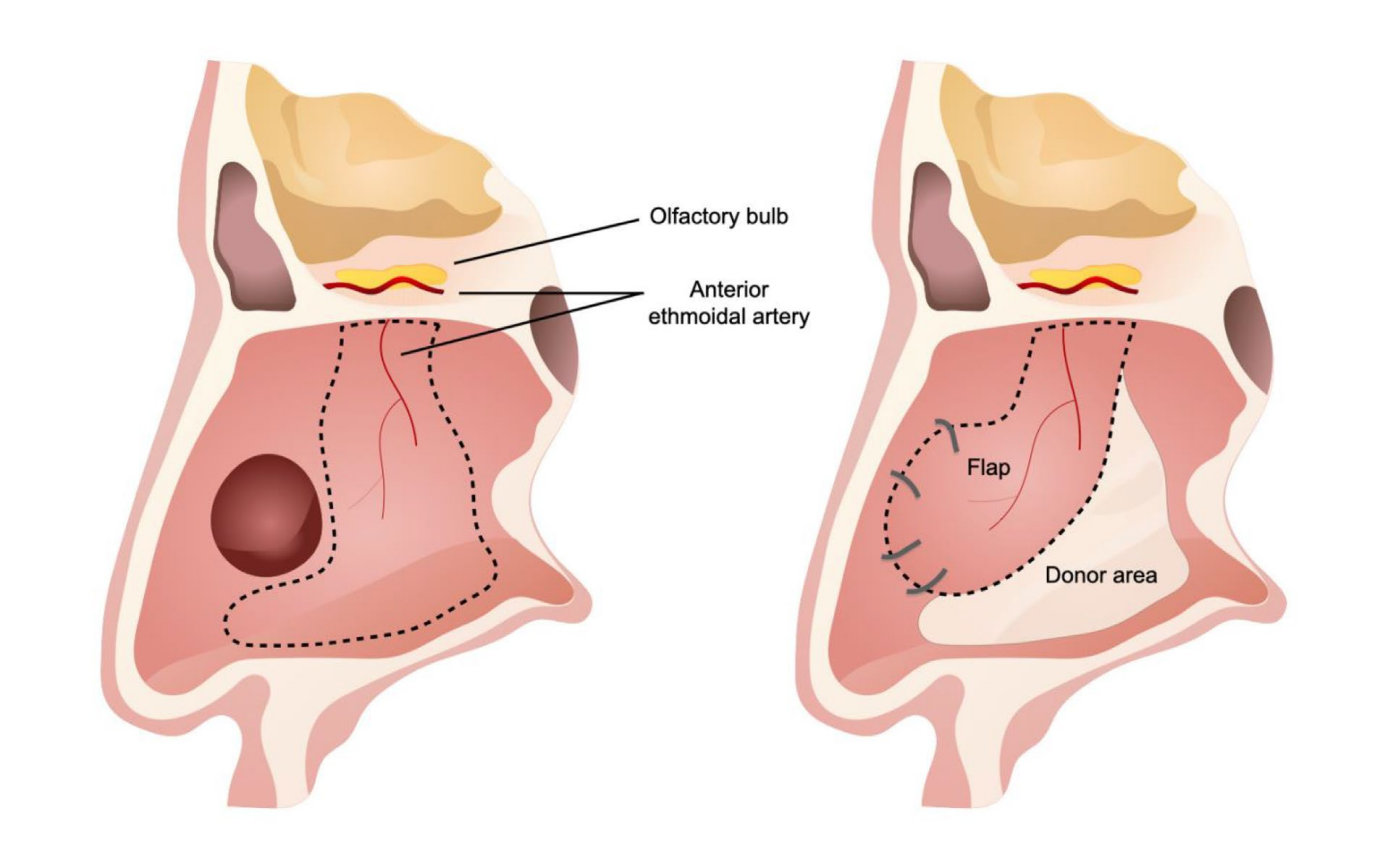
Anterior Ethmoidal Artery (AEA) flap technique illustration. [Color figure can be viewed in the online issue, which is available at www.laryngoscope.com]

Employing the PICO(TS) (Population/Problem, Intervention, Comparator, Outcome, Timing, Setting) framework for systematic reviews, the population was pediatric or adult patients with NSPs. The intervention was endoscopic repair of NSPs with the AEA flap. While there was no predetermined direct comparator, a post hoc exploratory analysis was performed to compare the success of AEA flaps with and without interposition grafts. The primary outcome was the rate of successful complete NSP closure at follow‐up. For timing and setting, the duration of follow‐up and type of practice setting were inconsistently reported. Patient demographics and NSP characteristics data were collected. When reported, both AP and vertical dimensions were extracted; when only one measurement was provided, it was recorded as the maximal reported diameter.

Data were analyzed and descriptive statistics were performed in Microsoft Excel and GraphPad Prism (version 9.2.0; GraphPad Software, San Diego, CA, USA). Success was defined as complete NSP closure at follow‐up; cases with residual or recurrent perforation postoperatively or flap necrosis were delineated as failures. Failures were categorized as ‘early’ if they occurred ≤ 3 months after NSP repair and ‘delayed’ if they occurred at > 3 months of follow‐up [13]. Due to the heterogenous reporting trends in the literature, a meta‐analysis could not be performed. A subgroup analysis to evaluate the impact of NSP size on closure success rate was performed; however, the unavailability of patient‐specific data for a majority of patients precluded a meaningful analysis.

## 3 Results

### 3.1 Study Selection

The initial search revealed 2838 partially overlapping results, of which 2169 were unique (Figure 1). In total, 2005 studies were deemed outside the scope of the review and were excluded. Full‐text analysis was performed for 164 studies. Sixteen studies were selected for data extraction and included for analysis.

### 3.2 Study Characteristics

All 16 studies described cases of NSP repair with the AEA flap technique, with some studies reporting technique modifications. Included studies were published between 2011 and 2026 (Table 1). Fourteen studies were designated as case series, one two‐patient series that also incorporated a cadaveric component, and one retrospective cohort study. Thirteen studies were single institution, and 3 were multi‐institutional. Level of evidence ranged from level 3 to 5 based on the Oxford Centre for Evidence‐Based Medicine guidelines [10].

**TABLE 1:** Summary and characteristics of the included studies.

First author publication year	Surgical technique	Study type	Number of patients	M/F	Patient age	Mean size of septal perforation	Septal perforation size range	Successful closure	Postoperative complications	Follow‐up time	Bias rating	Level of evidence	MINORS criteria score^a^
Castelnuovo 2011	AEA flap	Case series	11	7/4	Mean 41.6 (31–57)	15 mm (AP diameter)	10–25 mm	11/11	None	12–51 months	Good	4	11/16
Gras‐Cabrezio 2016	AEA flap	Case series; cadaver	5 cadavers; 2 patients	2/0	35, 42	N/A	N/A	2/2	None	6–12 months	Fair	5	7/16
Haussler 2018	AEA flap	Case series	5	N/A	N/A	12.8 mm (largest diameter)	8–23 mm	4/5	None	6–12 months	Fair	5	7/16
Cavada 2019	AEA flap with inlay graft of collagen matrix	Case series	13	9/4	Mean 50 (23–71)	18 mm (largest diameter)	3–35 mm	13/13	*N* = 1 with crusting, *N* = 1 required steroid injection of bulky AEA flap with good resolution	3 months (for all patients)	Good	4	11/16
Rusetsky 2020	Cross‐septal returned flap (CSRF), extension of AEA flap by widening flap posteriorly and laterally	Case series	9 (6 pediatric, 3 adult)	N/A	N/A	Sagittal axis: 17.7 mm Vertical axis: 16.9 mm	Sagittal: 7–30 mm Vertical: 5–30 mm	8/9	None	Mean 23 months (12–31 months)	Good	4	10/16
Rusetsky 2020	AEA flap with Inverted Edges Technique (IET), CSRF for bilateral closure	Case series	17 (pediatric)	N/A	Mean 13.8 (9–17)	Sagittal axis: 17.8 mm Vertical axis: 13.2 mm	Sagittal: 7–30 mm Vertical: 5–30 mm	AEA: 0/1 AEA + IET: 10/10 CSRF: 5/6	AEA + IET: *N* = 1 with crusting	Mean 14 months (4–31 months)	Good	4	10/16
Nassif 2021	AEA flap with hinge flap (posterosuperior mucosa wedge as contralateral surface coverage)	Case series	3 (pediatric)	N/A	Mean 12.3 (12–13)	10 mm (AP diameter)	6–12 mm	3/3	None	6–12 months	Fair	4	8/16
Santamaria‐Gadea 2021	Extended AEA (eAEA) flap	Case series	6	3/3	Mean 46.5 (36–66)	35 mm (largest diameter)	30–45 mm	6/6	None	Mean 11.3 months (8–16 months)	Good	4	8/16
Garaycochea 2022	eAEA flap	Case series	3	3/0	Mean 49.7 (43–55)	27 mm (not specified)	19–32 mm	3/3	None	Mean 11 months (8–13 months)	Fair	4	10/16
Zocchi 2022	AEA flap	Case series	32	16/16	Mean 47 (14–75)	14.8 mm (AP diameter)	4–30 mm	26/32	*N* = 4 crusting, *N* = 6 nasal obstruction, *N* = 2 epistaxis, *N* = 2 flap necrosis, *N* = 1 revision to reduce bulky flap	Median 38 months	Good	3	11/16
Bayoumi 2023	AEA flap with/without cartilage graft	Case series	18	6/12	Mean 42.4 (25–72)	13.6 mm (largest diameter, horizontal or vertical)	4–28 mm	17/18 (13/14 with graft, 4/4 without graft)	*N* = 1 epistaxis	Mean 7.3 months (6–9 months)	Good	4	11/16
Tumlin 2023	AEA flap with inlay graft of collagen matrix	Case series	41	19/22	Mean 42.5 (14–65)	22 mm (AP diameter)	5–45 mm	30/41	None	Mean 9 months (3.6–16.4 months)	Good	3	9/16
Almahbub 2024	AEA flap with tunnel technique	Case series	18	12/6	Mean 34.9 (SD: 14)	15 mm (largest diameter, horizontal or vertical)	5–36 mm	18/18	*N* = 3 crusting, *N* = 1 nasal obstruction due to thickened flap, *N* = 2 epistaxis *N* = 1 recurrent perforation at 12 month follow‐up	Mean 7 months (SD: 5.2 months)	Good	4	9/16
Alobid 2024	Boot‐on‐donut: eAEA flap (Boot) + Inverted edges flap (Donut)	Case series	18	11/7	Mean 39.2 (18–62)	Antero‐posterior: 23.4 mm; Superior–inferior: 16.6 mm	Antero‐posterior: 10–34 mm; Superior–inferior: 10–23 mm	18/18	None	Mean 18.7 months (6–42 months)	Good	4	11/16
Rusetsky 2024	AEA flap vs. Bilateral repair (Inverted edge technique with posterior septal artery flap)	Retrospective cohort	47 AEA 52 BLR	AEA: 16/31 BLR: 13/39	AEA: Mean 33 (SD 10.8) BLR: Mean 37.1 (SD 12.1)	AEA: 14.6 mm sagittal plane BLR: 18.3 mm sagittal plane	AEA: 5–30 mm BLR: 3–40 mm	AEA: 39/47 BLR: 48/52	Crusting and nasal obstruction (number of patients not reported)	12–24 months	Good	3	15/24
Tumlin 2025	AEA flap with cadaveric rib cartilage graft	Case series	6	2/4	43.5 (SD: 13.7)	13.0 mm (largest diameter)	7–30 mm	6/6	*N* = 5 nasal obstruction, *N* = 1 flap hypertrophy requiring debridement	Mean 9.5 months (2–22 months)	Good	4	9/16

Abbreviations: AEA, anterior ethmoidal artery; AP, anterior–posterior; BLR, bilateral repair; CSRF, cross septal return flap; eAEA, extended anterior ethmoidal artery; IET, inverted edges technique; MINORS, Methodological items for non‑randomized studies; NSP, nasal septal perforation; SD, standard deviation.

^a^ Of note, in regards to MINORS criteria score, the maximum score is 16 for non‐comparative studies and 24 for comparative studies. For non‐comparative studies, good quality studies have scores of 14–16, moderate quality studies score 8–13, and poor quality studies score < 8. For comparative studies, scores of 15–22 suggest moderate quality.

### 3.3 Clinical Data and Outcomes

Across the 16 studies, clinical data was available for 249 patients with NSPs who underwent a version of the AEA flap (original or modified). Of the 249 patients, 223 were adults and 26 were pediatric. For adults, 175 patients underwent the original AEA flap with (*N* = 74) or without (*N* = 101) an interposition graft. All pediatric patients and 48 adult patients underwent various modified AEA flap techniques.

For adult patients that underwent the original AEA flap, 44.0% were male (*N* = 77); data regarding patient sex was not available for the pediatric population. Across studies, mean age for the adult population was 41.0 and 13.9 years for the pediatric population. Table 2 shows frequencies of presenting symptoms in adult and pediatric NSP patients. In adults, crusting was the most commonly reported symptom in 38% of patients (*N* = 52), followed by nasal obstruction in 35.8% of patients (*N* = 49), epistaxis in 22.6% (*N* = 31), and whistling in 6.7% (*N* = 9). In pediatric patients, the most common symptom was crusting in 28.6% of patients (*N* = 14), followed by nasal obstruction in 26.5% (*N* = 13), epistaxis in 22.4% (*N* = 11), and whistling in 20.4% (*N* = 10).

**TABLE 2:** Symptoms reported for adult and pediatric patients with nasal septal perforations.

	Adults *N* = 137 (%)	Pediatric *N* = 49 (%)
Crusting	52 (38.0)	14 (28.6)
Nasal obstruction	49 (35.8)	13 (26.5)
Epistaxis	31 (22.6)	11 (22.4)
Whistling	9 (6.7)	10 (20.4)
Rhinorrhea	3 (2.2)	0 (0)
Dryness	2 (1.5)	0 (0)
Saddle nose deformity	0 (0)	1 (2.1)

*Note:* Of note, *N* is variable for the groups given that patients may have reported > 1 symptom.

Table 3 shows frequencies of NSP etiologies in adult and pediatric patients. In adults, iatrogenic causes accounted for nearly half of the NSPs (48.8%, *N* = 82). Other commonly reported causes in adults included idiopathic (38.1%, *N* = 64), intranasal drug use (5.4%, *N* = 9), and trauma (5.4%, *N* = 9). For pediatric patients, most common etiologies included intranasal corticosteroid or decongestant use (30.8%, *N* = 8) and nasal trauma (30.8%, *N* = 8). Nasal cautery was the reported cause for 15.4% of pediatric patients (*N* = 4). In adults, other less common causes of NSPs were nasal cautery and chemical occupational exposure. In pediatric patients, other less common causes of NSPs were chemical exposure, autoimmune disease, and foreign body.

**TABLE 3:** Etiologies of nasal septal perforations for adult and pediatric patient populations.

	Adults *N* = 168 (%)	Pediatric *N* = 26 (%)
Iatrogenic	82 (48.8)	0 (0)
Idiopathic	64 (38.1)	3 (11.5)
Intranasal corticosteroid (INCS)/decongestant use	0 (0)	8 (30.8)
Trauma	9 (5.4)	8 (30.8)
Nasal cautery	3 (1.8)	4 (15.4)
Drug use	9 (5.4)	0 (0)
Other^a^	1 (0.6)	3 (11.5)

^a^ Other category includes chemical exposure, related to treatment of autoimmune disease, and foreign body.

The reported mean NSP size ranged from 12.8 to 22 mm (minimum 3 mm and maximum 45 mm). Postoperative follow‐up time ranged from a minimum of 2 months to a maximum of 51 months across all studies. For all 249 patients, NSP closure success rate was 88.0%. Overall, for adult patients, NSP closure success rate was 84.6% (*N* = 148/175) for the AEA flap with or without an interposition graft (Figure 3). Adults who underwent a modified AEA flap had a reported NSP closure rate of 100% (*N* = 48/48) with 1 patient having recurrent perforation after 12 months of complete closure. Notably, this modified AEA flap group included heterogeneous techniques with numerous modifications further characterized in the discussion section. There was no significant difference in closure success rate when comparing patients with and without an interposition graft (83.8% (*N* = 62/74) vs. 85.1% (*N* = 86/101), respectively, *p* = 0.81, Cramer's *V* = 0.02) during a post hoc analysis. Across the 27 adult patients who did not have successful NSP closure, 4 patients had delayed failure with residual perforations and 2 patients had early failure with flap necrosis [14]; timing of failure was not reported for 21 patients [8, 15, 16, 17].

**FIGURE 3:**
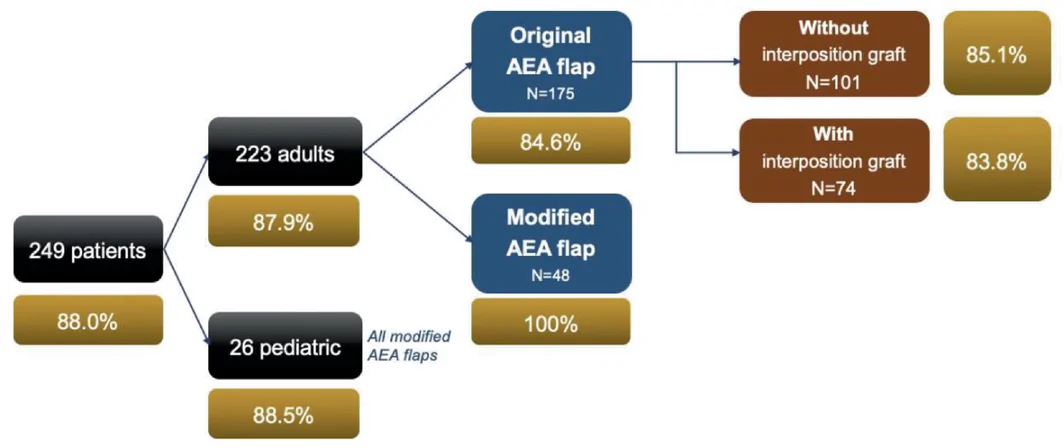
Successful closure rates reported for anterior ethmoidal artery (AEA) flaps for adult and pediatric patient populations and technique variations. [Color figure can be viewed in the online issue, which is available at www.laryngoscope.com]

Regarding postoperative complications, 62.5% of studies (*N* = 10/16) reported no complications throughout their respective follow‐up periods. The most commonly reported postoperative complication in adults was nasal obstruction (*N* = 12), followed by crusting (*N* = 8) and epistaxis (*N* = 5). Three patients with successful closure required interventions to reduce flap bulk postoperatively. No studies reported postoperative infections.

For pediatric patients, closure success rate was 88.5% (*N* = 23); all patients underwent a modified AEA flap approach. The three failed cases had early failure with residual perforations at initial follow‐up. Postoperative crusting was reported for one pediatric patient; no other complications were reported.

## 4 Discussion

This systematic review presents the first comprehensive analysis of all published literature on the efficacy of the AEA flap and its modifications for endoscopic NSP repair.

### 4.1 NSP Symptoms and Size

Across the included studies, the most commonly reported symptoms of NSP for both adult and pediatric patients were nasal obstruction and crusting. Regarding NSP etiologies, iatrogenic and idiopathic causes were most prevalent in adults. Intranasal corticosteroid or decongestant use and trauma were the most commonly reported etiologies among pediatric patients.

While manifestation of symptoms is often contingent upon NSP location, with more anterior perforations being symptomatic, surgical repair options and success are often contingent upon NSP size. Generally, NSPs ≤ 1.5–2 cm are reported to have higher closure rates (92% vs. 90% for larger NSPs) [7]. Larger NSPs, often defined as > 2 cm, are challenging to close due to limited tissue availability, increased mucosal tension, and reduced vascularity [18]. The AEA flap, initially described by Castelnuovo, was used to repair NSPs up to 2–2.5 cm [9].

NSP sizes were heterogeneously reported as either the greatest diameter, antero‐posterior (AP) or vertical diameter, with few studies providing both diameter measurements (Table 1). For NSP repairs with the original AEA flap technique without interposition grafts, the upper limit of NSP size reported was 2.5–3.0 cm [9, 14, 15, 16, 17, 19]. For NSP repairs with the original AEA technique with an interposition graft, the upper limit of NSP size was 3.0–4.5 cm [8, 16, 20]. For NSP repairs with a modified AEA flap technique (e.g., eAEA flap) with or without interposition graft, the upper limit of NSP size increased to 3.5–4.5 cm [21, 22, 23, 24, 25, 26, 27]. These studies highlight the utility of the AEA flap, with and without modifications, for repairing a wide range of NSP sizes.

### 4.2 Original Surgical Technique

As first described by Castelnuovo, the AEA flap is a unilateral, superiorly‐based transpositional flap that contains both perichondrium and mucoperiosteum (Figure 2). First, curettage of the anterior edge of the perforation is performed to promote a minor amount of bleeding to assist integration of the flap after suturing [9]. The posterior aspect of the perforation marks the anterior most edge of the AEA flap. The initial incision forms the posterior border of the flap and is vertically fashioned along the septum 0.5–1.0 cm posterior to the septal projection of the middle turbinate axilla [9, 19]. The posterior vertical incision is carried to the nasal floor, and along the junction of the hard and soft palate, until reaching the posterior aspect of the lateral wall of the inferior meatus. The incision is continued anteriorly to the posterior edge of the NSP, and kept inferior to the nasolacrimal duct orifice. The incision is then extended medially along the nasal floor to the posteroinferior edge of the NSP. The AEA‐based flap is then elevated, and rotated about its superiorly‐based pedicle as it is advanced and sutured to the mucosa around the NSP's perimeter.

The original AEA flap technique is viewed as a favorable option for several reasons. First, the AEA flap is harvested unilaterally, leading to minimal donor site morbidity, and preservation of the contralateral nasal septal and floor mucosa allows for another future repair should the initial repair attempt fail [14]. Unilateral flap harvest also decreases surgical dissection time and could decrease the risk of enlarging the NSP or creating a new NSP [16, 17]. Lastly, this flap is a one‐stage procedure, obviating a return to the operating room. While the AEA flap has numerous advantages, repairing NSPs greater than 2–3 cm is a potential limitation depending on NSP location and orientation, especially for NSPs with a greater vertical dimension.

While the AEA flap has been presumed to be axially based on septal perforators, a recent cadaveric study by Donaldson et al. suggested that its blood supply may be random [28]. They demonstrated in 10 cadavers (20 sides) that AEA branches coursed diagonally or horizontally from the cribriform plate along the septum. Since AEA flap septal incisions are made vertically, these AEA perforators will be divided, eliminating the axial supply. Because of this, presuming a 3:1 length: width ratio, they suggested the AEA flap base width should be 2.0–2.3 cm on average to ensure adequate perfusion [28].

Four studies implemented the original AEA technique with inlay of various collagen grafts (DuraMatrix, AlloDerm, and Biodesign sinonasal grafts), tragal cartilage or cadaveric rib grafts [8, 16, 20, 29]. Grafts are generally included to serve as a scaffold for the migration of respiratory mucosa and provide an additional layer of protection, but whether they augment NSP repair remains to be studied directly.

### 4.3 AEA Flap Technique Variations

A myriad of modifications to the original AEA technique have also been described to address NSP size limitations, though no comparative studies have been published comparing the different techniques (Table 4). The extended AEA flap (eAEA) technique was developed for the repair of larger NSPs > 2 cm [24]. The eAEA flap includes the mucosa of the sphenoid rostrum, from the choanal arch down to the nasal floor, and the entire inferior meatus mucosa to its posterior limit, through the hard and soft palate junction [24]. This technique has facilitated the successful repair of NSPs 3–3.5 cm in size, with minimal morbidity. Another modification involves elevating and inverting the septal mucosa above and/or below the NSP, then turning this mucosa in through the NSP to provide partial or complete contralateral NSP coverage [26]. Whether these turn‐in flaps improve success rates of NSP closure requires further study. In pediatric populations, Rusetsky et al. also described the inverting edges modification, as well as what they termed the cross‐septal returned flap (CSRF), which has also been used for adult patients [21, 22]. The CSRF technique, which necessitates a larger AEA flap than Castelnuovo's original flap, involves advancing the AEA flap to the contralateral side of the nasal cavity and covering both sides of the perforation with the CSRF; once the AEA flap is elevated, a vertical transfixion incision of the septum 1–3 mm anterior to perforation is made, and the flap is guided to cover the perforation on the ipsilateral side then through this vertical incision to the contralateral nasal cavity, turned backwards, sutured along the perimeter of the perforation [21, 22].

**TABLE 4:** Summary of the modifications to the original anterior ethmoidal artery flap technique reported in the literature.

Study	AEA flap modification
Adult patient population	
Rusetsky et al. (2020)	Cross septal returned flap (CSRF): flap tunneled through septum to cover both sides in order to achieve a simplified bilateral closure
Santamaria‐Gadea et al. (2021)	Extended AEA (eAEA) flap with mucosa from nasal floor, inferior meatus, and sphenoid rostrum
Garaycochea et al. (2022)	Extended AEA (eAEA) flap combined with a zigzag flap‐incision and inverted edges flap
Almahbub et al. (2024)	Tunnel Technique: a mucosal peri‐perforation flap used is as a tunnel around the perforation to secure the AEA flap directly against exposed cartilage
Alobid et al. (2024)	Boot‐on‐Donut Technique: extended AEA (boot) allows vascularized coverage and inverted edges (donut) reaches superior edge of large perforations/mucosal resurfacing from other side
Pediatric patient population	
Rusetsky et al. (2020)	Cross septal returned flap (CSRF): flap tunneled through septum to cover both sides in order to achieve a simplified bilateral closure
Rusetsky et al. (2020)	Inverted edges technique (IET): inverted edges of mucoperichondrium of perforation sutured prior to AEA flap affixation
Nassif et al. (2021)	AEA flap with hinge flap: wedge of mucosa at posterosuperior aspect of defect as a hinge flap, pedicled anteroinferiorly at posterosuperior margin

### 4.4 Outcomes of Repair With AEA Flaps

NSP closure success rate was 84.6% for all adult patients who underwent the original AEA flap with or without an interposition graft. There was no statistically significant difference in NSP closure success rates between those with or without an interposition graft (85.1% vs. 83.8%, respectively). Across the 48 patients who underwent various modified AEA flaps, the reported NSP closure rate was 100% with one case of recurrent perforation at follow‐up after 12 months of complete closure. These results must be interpreted with caution due to the heterogeneous technique modifications included in this group. The above aggregate results are also limited by the lack of patient‐specific data such as NSP size for nearly 75% of adult patients and variable postoperative follow‐up durations. Future studies are needed to determine whether AEA flap modifications improve NSP repair success and require more rigorous study designs and consistent follow‐up durations.

Only one study on AEA flap success reported potential risk factors of failure [8]. Tumlin et al. found that in 41 AEA flap repairs, older age, active smoking, diabetes mellitus, and history of intranasal substance use were significantly associated with NSP repair failure. Interestingly, while mean NSP size was 2.5 cm for failed NSP repair versus 2.1 cm for those with successful closure, this size difference was not statistically significant. Of note, they reported that 11 of 12 failed repairs had interposition grafts placed [8].

### 4.5 Pediatric Population

Surgical repair of NSPs in pediatric patients is often deferred until adolescence when nasal growth nears completion [23]. Although NSP repair is uncommon in children, the AEA flap has shown promise in pediatric patients and is preferred over open approaches to decrease the risk of scarring and avoid disturbing nasal tip support. Across three studies, 26 pediatric patients underwent NSP repair with modified AEA flap techniques [21, 22, 23].

Rusetsky et al. published the first and largest cohort of pediatric patients (*N* = 17) who underwent endoscopic NSP repair with modified AEA flap techniques, including the inverted edges technique or the CSRF technique [21]. Patients ranged from 8 to 17 years old, and NSP size upper limit was 2.5–3.0 cm [21]. Successful closure at 4–31 months postoperatively was reported in all patients who underwent the inverted edges technique and in 83% of patients who underwent CSRF technique [21]. In a subsequent study, Rusetsky et al. reported a similar successful NSP closure rate of 83% in pediatric patients undergoing the CSRF technique with residual perforation in one patient at 12–31 months follow‐up [22]. Another study by Nassif et al. used a modified AEA hinge flap technique for 3 adolescents with 6–12 mm NSPs and reported successful closure and symptom resolution in all patients at 6–12 months postoperatively [23].

### 4.6 Comparing AEA Flaps Versus Other Septal Perforation Repair Techniques

Surgical techniques for NSP repair include endoscopic and open approaches. The current synthesis of the literature demonstrates closure rates for the AEA flap that are comparable or superior to those of other NSP repair strategies. In line with this review's findings, Fermin et al. published a systematic review showing no significant differences in closure rates between open and endonasal approaches (89.4% vs. 90.9%, respectively, with *N* = 1591 patients across 64 studies) [7]. Another systematic review on endoscopic NSP repair only found that endoscopic techniques demonstrated an overall success rate of 90% (329 patients across 20 studies) [5]. The AEA flap demonstrated similar success rates to prior reports of open and endoscopic NSP repair, and should therefore be viewed as a viable option for repair of anterior NSPs, particularly those that are up to 2.5 cm in size for the original AEA flap and larger NSPs with modified AEA flaps.

### 4.7 Postoperative Complications

Notably, 60% of studies reported no postoperative complications throughout their respective follow‐up periods. The most commonly reported postoperative complications in adults were crusting, followed by nasal obstruction and epistaxis. Three patients who underwent the original AEA flap with successful closure were reported to require intervention in order to reduce flap bulk [14, 20, 29]. One patient who underwent AEA flap with collagen graft experienced nasal obstruction, which resolved with triamcinolone steroid injection of the bulky flap [20]. The second patient with a bulky flap had undergone successful closure with the traditional AEA flap and had requested revision surgery [14].

### 4.8 Study Limitations

A key limitation was the heterogeneous reporting of outcomes across the included studies. Follow‐up durations used to evaluate the success rate of closure were highly variable, ranging from 2 to 51 months postoperatively. Additionally, many authors did not clearly define closure success. Postoperative complications were inconsistently reported, as well. Technique‐specific outcomes were also challenging to determine and compare due to heterogeneity in techniques and data reporting. Due to lack of patient‐specific data reported by studies, mean values could not be calculated for several variables, especially NSP size and follow‐up duration. A subgroup analysis was performed to evaluate the impact of NSP size on success rate; however, patient‐specific data was only available for 30% of adult patients (*N* = 63) with only three patients having NSP closure failure, precluding a meaningful analysis. Given lack of adequate amount of patient‐specific data, subgroup analyses were not feasible. If a meta‐analysis were feasible, assessing the impact of NSP size and utilization of various interposition grafts on closure success rate, as well as identifying factors that may be associated with early vs. delayed NSP repair failure would be valuable. A potential avenue for sensitivity analysis would be a leave‐one‐out analysis to assess the impact of each study individually on the outcomes of a meta‐analysis. Another limitation was study quality, with all included studies having been case series or retrospective cohorts, with no prospective studies. Although the search strategy was designed by a librarian, it was not peer‐reviewed which presents a potential limitation. Furthermore, the moderate inter‐rater agreement (Cohen's kappa = 0.46) is another limitation of this study. Lastly, there was a lack of long‐term functional outcomes, objective measures, and standardized symptom assessments in the literature for patients undergoing NSP repair with AEA flaps. It is critical that future investigations employ prospective data collection, report standardized outcome measures (e.g., symptoms, quality‐of‐life, and endoscopic findings), and have consistent long term follow‐up which would help further validate the AEA flap's efficacy for NSP repair.

## 5 Conclusions

Based on systematic review, the AEA flap has yielded an overall NSP closure rate of 84.6%, similar to prior reports of both open and endoscopic NSP repair techniques. The AEA flap is a valuable reconstructive option for adult and select pediatric NSP patients.

## Funding

The authors have nothing to report.

## Conflicts of Interest

D.B.S.—consulting (Sanofi, Aerin Medical, Integra). The other authors declare no conflicts of interest.

## Supporting information


**Data S1:** Search Strategy for: anterior ethmoid artery flap for septal perforation.

## Data Availability

The data that supports the findings of this study are available in the Supporting Information of this article.
