# Supplementary material for: Success of Anterior Ethmoidal Artery Flaps for Nasal Septal Perforation Repair: A Systematic Review
Source: Laryngoscope. 2026 Jul 1;136(11):4638–49. doi: 10.1002/lary.70719 (PMC13669966; doi:10.1002/lary.70719)
Supplement: Supplementary file 1 [file LARY-136-4638-s001.docx]

**Supporting Information**

**Search Strategy for**: anterior ethmoid artery flap for septal perforation

**Initial PRISMA Numbers**

Records Identified from Databases: 2,565

Duplicate Records Removed (automatically with Covidence): 454

Records Uploaded for Title/Abstract Screening: 2,111

**Databases and Strategies**

**Proposed Databases for Searching**: MEDLINE, Embase, CENTRAL, and Web of Science

| Ovid MEDLINE(R) ALL 1946 to November 26, 2024 | | |
| --- | --- | --- |
| **Date Searched** | 11/27/2024 | |
| **Concept** | **Search String** | **Results** |
| **1** | ((nasal septum OR nasal septal OR nasoseptal OR nasoseptum) ADJ4 (perforat* OR closure)).ti,ab,kf. OR Nasal Septal Perforation/ | 902 |
| **2** | ((anterior ethmoid* OR AEA).ti,ab,kf. OR (Ethmoid Sinus/ AND Arteries/)) AND ((flap OR flaps).ti,ab,kf. OR Surgical Flaps/) | 47 |
| **Combo** | 1 OR 2 | 931 |
| **Limits** | AND NOT (exp Animals/ NOT Humans/)  AND English.lg.  AND Limit [n] to dt=20000101-20251231 |  |
| **Total** |  | 600 |

| Ovid Embase Classic+Embase 1947 to 2024 November 26 | | |
| --- | --- | --- |
| **Date Searched** | 11/27/2024 | |
| **Concept** | **Search String** | **Results** |
| **1** | ((nasal septum OR nasal septal OR nasoseptal OR nasoseptum) ADJ4 (perforat* OR closure)).ti,ab,kf. OR Nose Septum Perforation/ | 1,904 |
| **2** | ((anterior ethmoid* OR AEA).ti,ab,kf. OR (Anterior Ethmoidal Artery/)) AND ((flap OR flaps).ti,ab,kf. OR exp Surgical Flaps/) | 70 |
| **Combo** | 1 OR 2 | 1,954 |
| **Limits** | AND NOT (exp Animal/ NOT Human/)  AND English.lg.  AND Limit [n] to dc=20000101-20251231 |  |
| **Total** |  | 1,417 |

| Ovid EBM Reviews - Cochrane Central Register of Controlled Trials October 2024 | | |
| --- | --- | --- |
| **Date Searched** | 11/27/2024 | |
| **Concept** | **Search String** | **Results** |
| **1** | ((nasal septum OR nasal septal OR nasoseptal OR nasoseptum) ADJ4 (perforat* OR closure)).ti,ab,kw. OR Nasal Septal Perforation/ | 17 |
| **2** | ((anterior ethmoid* OR AEA).ti,ab,kw. OR (Ethmoid Sinus/ AND Arteries/)) AND ((flap OR flaps).ti,ab,kw. OR Surgical Flaps/) | 0 |
| **Combo** | 1 OR 2 | 17 |
| **Limits** | AND NOT (exp Animals/ NOT Humans/)  AND English.lg.  2000-Current |  |
| **Total** |  | 15 |

| Web of Science Core Collection | | |
| --- | --- | --- |
| **Date Searched** | 11/27/2024 | |
| **Concept** | **Search String** | **Results** |
| **1** | TS=(("nasal septum" OR "nasal septal" OR "nasoseptal" OR "nasoseptum") NEAR/4 ("perforat*" OR "closure")) | 674 |
| **2** | TS=(("anterior ethmoid*" OR "AEA") AND ("flap" OR "flaps")) | 40 |
| **Combo** | 1 OR 2 | 701 |
| **Limits** | AND English Language  AND DOP=2000-01-01/2025-12-31 |  |
| **Total** |  | 533 |
